# Native Aortic Valve Endocarditis Caused by Gut Bacterial Translocation Following Gastroenteritis

**DOI:** 10.7759/cureus.90972

**Published:** 2025-08-25

**Authors:** Allison Molstad, Alicia Latta, Elizabeth Willis

**Affiliations:** 1 Internal Medicine, Wright-Patterson Medical Center, Dayton, USA; 2 Internal Medicine, Wright State University, Dayton, USA

**Keywords:** aortic endocarditis, bacterial translocation, echocardiography for infectious endocarditis, infectious gastroenteritis, native valve endocarditis, viridans group streptococci

## Abstract

Native valve infective endocarditis (IE) refers to infection resulting from the seeding of bacteria onto the heart valves, usually in patients with one or more risk factors such as intravenous drug use, poor dentition or recent dental surgery, prior structural or valvular heart disease, or indwelling cardiac devices. Although viridans group streptococci (VGS) are a common cause of aortic valve endocarditis, it is uncommon for the infection to result from bacterial translocation across the intestinal mucosa during gastroenteritis. This case involves a 64-year-old female who presented with an acute onset of shortness of breath, weakness, nausea, vomiting, rigors, and night sweats lasting two to three weeks. She also reported a week of nonbloody diarrhea that has since resolved. A CT pulmonary angiogram revealed an acute pulmonary embolism (PE) in the right upper lobe without evidence of right heart strain. A transthoracic echocardiogram, obtained during the work-up for her PE, showed a mobile density on the right coronary cusp concerning for vegetation. Transesophageal echocardiogram confirmed a mobile density on the right coronary cusp of the aortic valve, consistent with a vegetation larger than 10 mm. Blood cultures grew *Streptococcus oralis/mitis*, and the patient was diagnosed with IE. She subsequently underwent early aortic valve replacement with cardiothoracic surgery. The source of the IE in this patient remains unclear. However, based on her history of a week of profuse, nonbloody diarrhea prior to any other symptom onset, it is likely that bacterial translocation from the gut during an acute episode of gastroenteritis was the source of the bacteremia leading to vegetation. While VGS are typical causes of IE, the infection usually originates from the oral cavity and less commonly from the lower GI tract. This patient had no recent dental procedures and no dental issues to suggest an oral route of infection. Her presentation with acute PE and subsequent diagnosis of native aortic valve endocarditis secondary to gastroenteritis was atypical, particularly as it occurred in a native valve without underlying risk factors.

## Introduction

Infective endocarditis (IE) is a rare but serious condition characterized by infection of the endocardial surface of the heart, most commonly affecting the cardiac valves. It typically arises from bacteremia, leading to colonization of the heart valves or endocardium and subsequent inflammation. The incidence of IE is estimated at three to 10 cases per 100,000 individuals annually, with a male predominance of approximately 2:1 [1].

IE is associated with predisposing risk factors such as intravenous drug use (IVDU), poor dentition or recent dental procedures, structural or valvular heart disease, and the presence of indwelling cardiac devices [2]. Additional risk factors include diabetes mellitus, hemodialysis, and, although less common in modern times, rheumatic heart disease [2]. Among these, IVDU is increasingly recognized as a major contributor, now accounting for nearly 10% of all IE cases [2].

The predominant causative organisms in IE are *Staphylococci*, *Streptococci*, and *Enterococci*, which together account for up to 90% of cases [2]. *Staphylococcus aureus* is the most frequently isolated pathogen, responsible for about 30% of cases [2]. Coagulase-negative staphylococci, although generally less virulent, are more commonly associated with prosthetic valve infections, indwelling devices, and community-acquired cases, comprising approximately 20% of infections [2]. Enterococci account for approximately 15-18% of cases [2]. Viridans group streptococci (VGS) are identified in about 20% of cases and are particularly associated with community-acquired infections [2]. When *Streptococcus gallolyticus *(formerly *Streptococcus bovis*) is isolated, it raises a strong suspicion for an underlying colonic carcinoma [2].

This case report presents a rare instance of IE in a patient with no identifiable risk factors, who developed IE following a GI illness, suggesting a possible GI source of bacteremia post-gastroenteritis.

## Case presentation

A 64-year-old female with a past medical history notable only for colon polyps presented to the emergency department with acute-onset shortness of breath after a short interstate flight. Associated symptoms included weakness, nausea, vomiting, rigors, and night sweats for the preceding two to three weeks. She denied chest pain or fever. Notably, she reported a self-limited episode of non-bloody diarrhea lasting approximately one week during her trip, which had resolved by the time of presentation (Table 1).

**Table 1:** Temporal sequence of events leading to the patient’s presentation

Timeline	Symptoms
Week 1	Gastroenteritis with non-bloody diarrhea, resolved by week 2
Weeks 2-3	Progressive systemic symptoms, including chills, rigors, nausea, and vomiting
Week 4	Presentation to the emergency department with shortness of breath

On physical examination, no cardiac murmurs were appreciated, pulmonary findings were unremarkable, and no abdominal tenderness was present. Initial laboratory workup in the ED revealed borderline leukocytosis with a left shift, including 21% band forms. A CT pulmonary angiogram demonstrated an acute pulmonary embolism (PE) in the right upper lobe pulmonary artery, without evidence of right heart strain. Further imaging with CT of the abdomen and pelvis showed splenomegaly.

**Figure 1:**
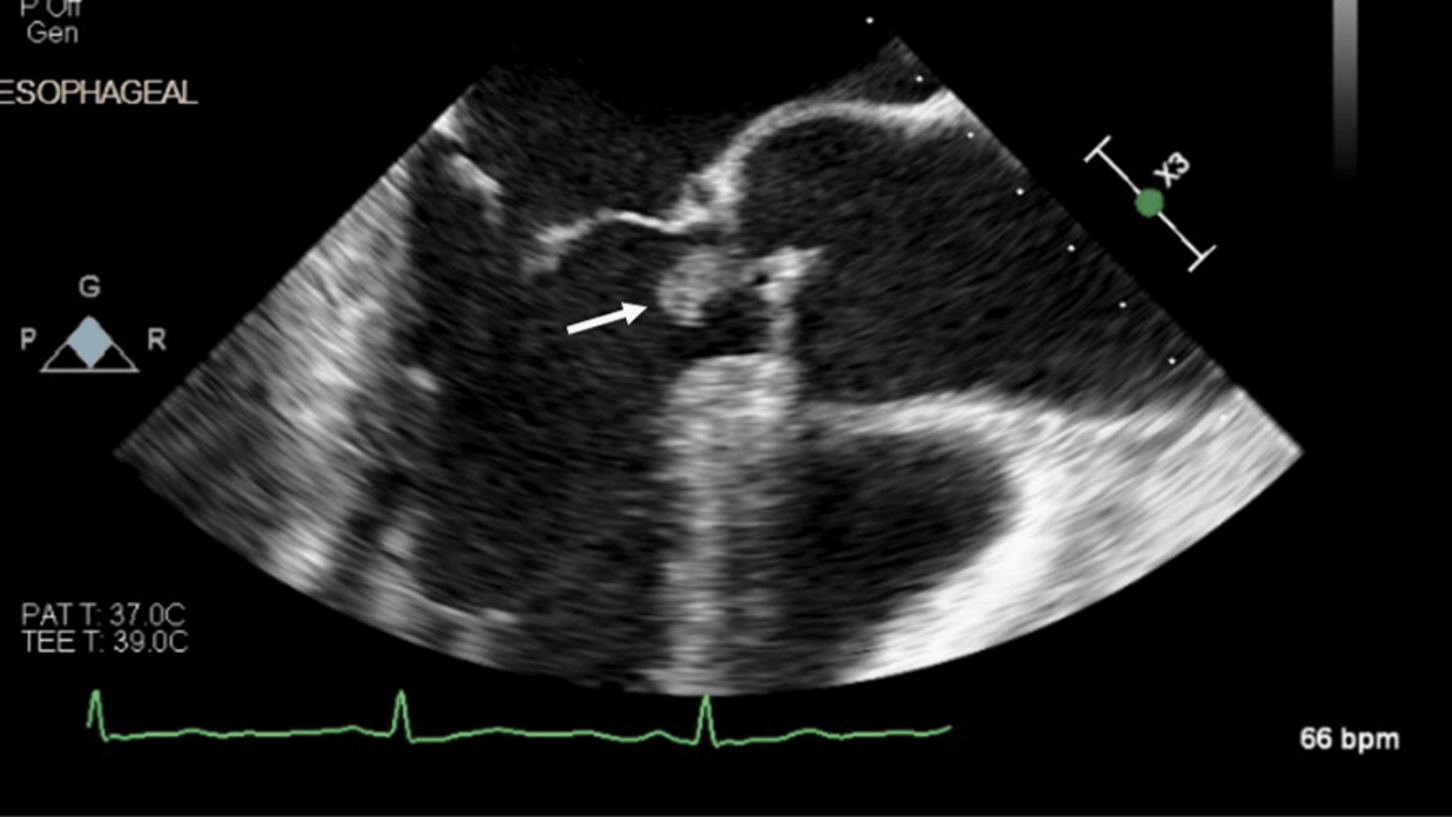
TEE showing a >10-mm mobile density on the noncoronary cusp in the mid-esophageal long-axis view TEE, transesophageal echocardiogram

**Figure 2:**
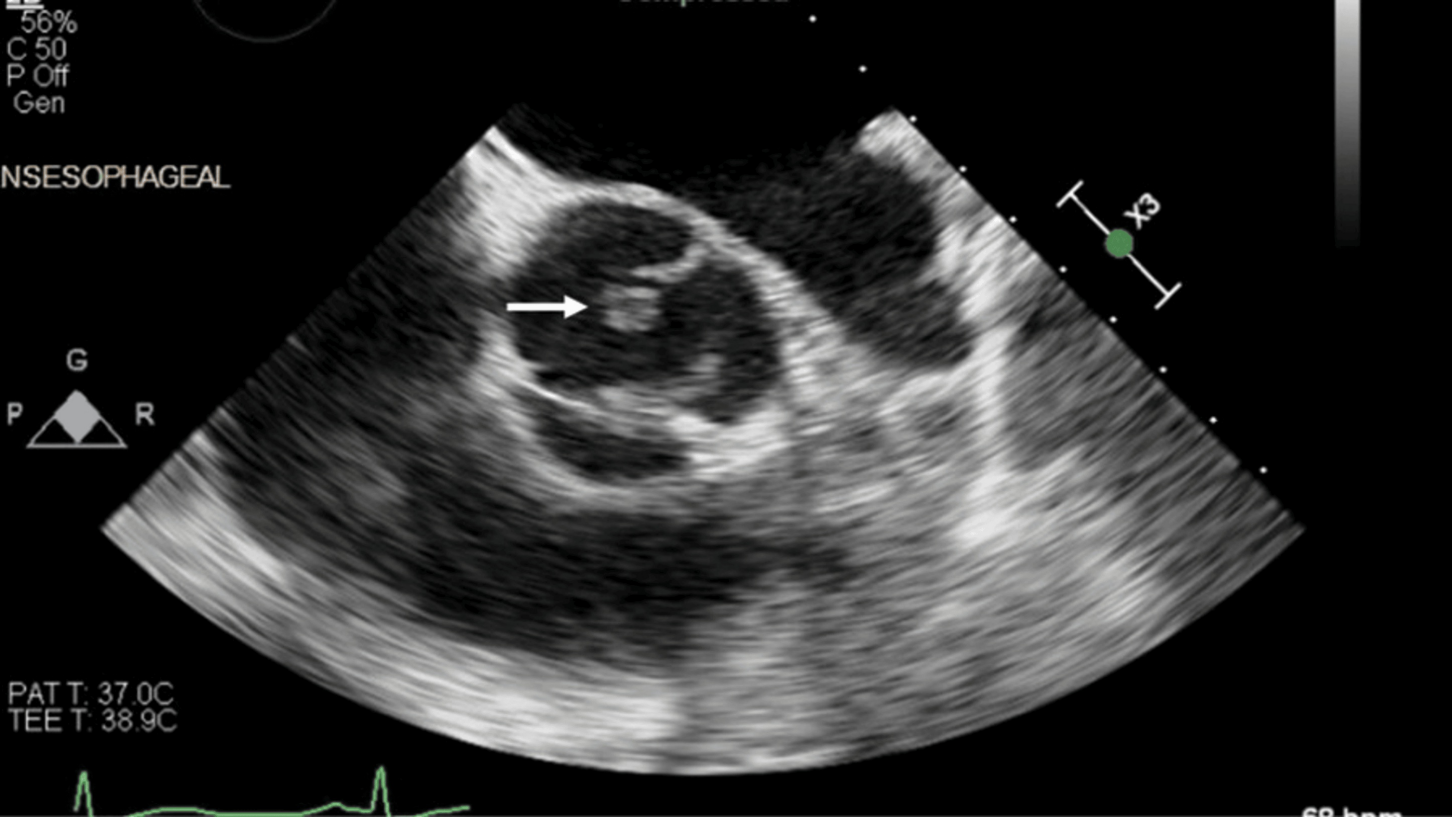
TEE in the mid-esophageal aortic valve short-axis view TEE, transesophageal echocardiogram

**Figure 3:**
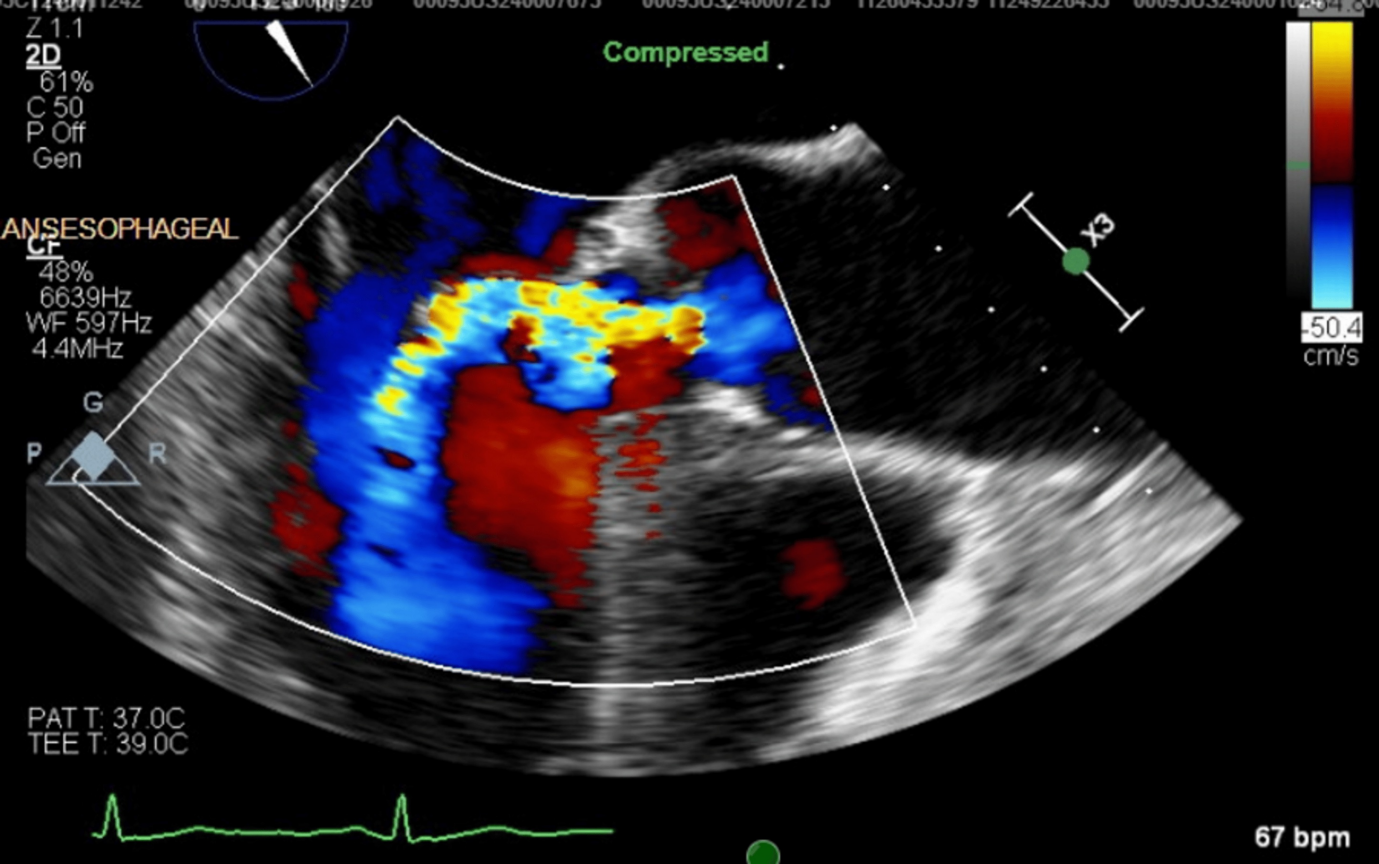
TEE color Doppler view showing aortic regurgitation TEE, transesophageal echocardiogram

As part of the routine evaluation for PE, a transthoracic echocardiogram was performed, which revealed a mobile echodensity on the right coronary cusp of the aortic valve, concerning for vegetation. Cardiology recommended additional imaging with a transesophageal echocardiogram (TEE), which confirmed a vegetation >10 mm on the non-coronary cusp of the aortic valve, associated with moderate aortic insufficiency (Figure 1, Figure 2, Figure 3).

The infectious disease team initiated empiric IV vancomycin. Blood cultures subsequently grew *Streptococcus oralis/mitis*, and the diagnosis of IE was established based on the 2023 Duke-International Society for Cardiovascular Infectious Diseases Criteria [2]. Given the size of the vegetation and associated valvular dysfunction, cardiology recommended cardiothoracic surgery evaluation. The patient underwent early aortic valve replacement without complications. She was treated with a six-week course of intravenous ceftriaxone, and follow-up blood cultures remained consistently negative.

On subsequent cardiology follow-up, the patient had no cardiac complications following aortic valve replacement.

## Discussion

IE remains a significant cause of morbidity and mortality, even in patients without traditional risk factors. Prompt identification and initiation of treatment are critical to improving outcomes. Classically, IE presents with insidious symptoms such as fever, chills, fatigue, headache, and generalized weakness, which are often ambiguous or nonspecific. In advanced cases, extensive valvular damage may lead to signs of left- or right-sided heart failure, depending on the valve involved. Fever (defined as a temperature >38°C) is present in up to 95% of patients with IE, while cardiac murmurs are documented in approximately 50% [3]. However, this patient remained afebrile throughout her hospital course and exhibited no murmurs, making the diagnosis more elusive. She also lacked the classic dermatologic stigmata of acute IE, such as Janeway lesions, Osler nodes, and splinter hemorrhages, which result from septic emboli. Although these findings are emphasized in clinical education, they occur in only about 10% of cases and are neither sensitive nor specific for IE [2]. Interestingly, the patient’s splenomegaly served as an important but subtle clue. Splenomegaly is a well-documented finding in IE and may result from reactive splenic hyperplasia due to systemic infection [4] or from septic embolization to the spleen from an aortic valve vegetation [5].

This case was particularly unusual in that the patient initially presented with acute PE, a symptom not typically associated with left-sided IE. The aortic valve vegetation was discovered incidentally during echocardiographic evaluation for PE. Only after this finding prompted further investigation did the patient reveal a history of subjective fevers, rigors, and night sweats, symptoms more typical of subacute endocarditis. This highlights a key diagnostic challenge: the initial low clinical suspicion for IE due to its atypical presentation and absence of classical findings. The case underscores the importance of obtaining a detailed clinical history and maintaining a high index of suspicion for “can’t-miss” diagnoses such as IE, even in the absence of traditional risk factors or textbook symptoms.

Another noteworthy aspect of this case is the coexistence of PE and IE. This raises an important clinical question: did these two pathologies occur coincidentally, or was one a direct consequence of the other? In right-sided IE, particularly involving the tricuspid valve, septic emboli to the pulmonary circulation are common complications. However, in left-sided IE, vegetations usually embolize to the systemic arterial circulation, resulting in infarcts or abscesses in organs such as the brain, spleen, kidneys, or mesenteric vasculature [5]. Without an abnormal intracardiac communication, such as a patent foramen ovale, arteriovenous malformation, or pulmonary arteriovenous fistula, direct embolization to the lungs from a left-sided vegetation is physiologically implausible. Given this patient’s aortic valve involvement, it is highly unlikely that the PE resulted from septic emboli. A more plausible explanation is that the systemic inflammatory response triggered by bacteremia and IE created a prothrombotic state, fulfilling Virchow’s triad of endothelial injury, hypercoagulability, and stasis. This systemic hypercoagulability likely led to venous thromboembolism and the acute PE that prompted the initial work-up. Paradoxically, the PE was the presenting complaint, while the IE was the far more serious underlying diagnosis. This case illustrates how systemic infection, even when atypical or subclinical, can predispose patients to thromboembolic complications and how incidental findings may unmask critical diagnoses.

The etiology of IE in this patient remains unclear, adding a unique dimension to the case. Notably, she lacked all conventional risk factors typically associated with IE [2]. A key detail was her report of a week-long episode of profuse, non-bloody diarrhea during a recent trip, which preceded the onset of systemic symptoms. This raises the possibility that an acute bout of gastroenteritis led to bacterial translocation across the intestinal mucosa, resulting in bacteremia and subsequent seeding of the aortic valve with vegetation formation. Bacterial translocation in the GI tract may occur through several mechanisms, including intestinal bacterial overgrowth, increased intestinal permeability due to trauma or inflammation, and host immunodeficiency [6]. In this case, the patient was not immunocompromised, ruling out that cause. Undiagnosed bacterial overgrowth remains a possible factor, but significant mucosal inflammation following enteritis likely increased intestinal permeability and allowed bacteria to enter the bloodstream. Prophylactic use of probiotics in intensive care settings has been shown to reduce the risk of sepsis, supporting the role of bacterial translocation in the development of bacteremia [7]. This aligns with the “gut origin hypothesis of sepsis,” which proposes that bacteremia and sepsis may originate from gut-derived pathogens, particularly following infection, surgery, or trauma [8].

Bacteremia is a rare complication of gastroenteritis, often presenting as prolonged fever, chills, and malaise without concurrent diarrhea, as in this case [9]. Sustained bacteremia can lead to severe complications such as endocarditis or infected aneurysms [9]. Although uncommon, bacterial translocation due to increased mucosal permeability from GI inflammation can result in systemic infection, ultimately causing clinical manifestations such as IE.

While VGS, such as *S. oralis/mitis*, are well-established causes of community-acquired IE, they are most commonly linked to the oral cavity [2]. This patient, however, had no recent dental procedures and demonstrated good oral hygiene, making an oral source unlikely. Although lower GI colonization by *S. oralis/mitis *is rare, it has been documented. In this case, the temporal relationship between GI symptoms and systemic illness supports the GI tract as a plausible, albeit uncommon, source. This highlights that, in the absence of traditional risk factors, transient mucosal barrier disruption during gastroenteritis may serve as an entry point for pathogenic bacteria, leading to bacteremia and subsequent endocardial infection.

Although this patient lacked traditional risk factors for IE, she did have a history of colonic polyps, which may be a relevant consideration in the etiology of her infection. The association between colorectal neoplasia and *S. gallolyticus *(formerly *S. bovis*) is well-documented and has led to recommendations for colorectal cancer screening in patients with *S. gallolyticus *bacteremia or endocarditis. Although *S. oralis/mitis *is not traditionally included in this association, emerging evidence suggests that other members of the VGS may also play a role in colonic pathology [10]. Recent case reports and studies have identified *S. mitis* in particular in patients with colorectal cancer and precancerous polyps, suggesting a potential association [10]. While the mechanism remains unclear, hypotheses include bacterial translocation across neoplastic or inflamed colonic mucosa or possible modulation of local immune responses or microbiome balance. In this patient, the combination of *S. oralis/mitis *bacteremia, IE, and a history of colonic polyps raises the question of whether colonic pathology contributed to bacterial entry into the bloodstream [11-14].

## Conclusions

This patient’s presentation of acute PE with subsequent diagnosis of native aortic valve endocarditis secondary to GI bacterial translocation was atypical, particularly as it occurred in a native valve without underlying risk factors. This case highlights the importance of thorough clinical history-taking and maintaining a wide differential diagnosis with a high index of suspicion for “can’t-miss” conditions such as IE. It also underscores that risk factors are only part of the picture and that many diseases may occur in individuals regardless of risk status. Looking forward, it will be important to further explore the relationship between colonic malignancy and *S. mitis *bacteremia, mirroring established knowledge regarding *S. gallolyticus*.

## References

[1] Cahill TJ, Prendergast BD (2016). Infective endocarditis. Lancet.

[2] Yallowitz AW, Decker LC (2025). Infectious endocarditis. StatPearls [Internet].

[3] Murdoch DR, Corey GR, Hoen B (2009). Clinical presentation, etiology, and outcome of infective endocarditis in the 21st century: the International Collaboration on Endocarditis-Prospective Cohort Study. Arch Intern Med.

[4] Chapman J, Goyal A, Azevedo AM (2025). Splenomegaly. StatPearls [Internet].

[5] Santos EL, Lima AD, Dompieri L, Holanda AC, Aquino MA, Lopes RD (2018). Paradoxical embolism in a patient with aortic valve endocarditis: a case report. Cureus.

[6] Berg RD (1995). Bacterial translocation from the gastrointestinal tract. Trend Microbiol.

[7] Doudakmanis C, Bouliaris K, Kolla C, Efthimiou M, Koukoulis GD (2021). Bacterial translocation in patients undergoing major gastrointestinal surgery and its role in postoperative sepsis. World J Gastrointest Pathophysiol.

[8] Haussner F, Chakraborty S, Halbgebauer R, Huber-Lang M (2019). Challenge to the intestinal mucosa during sepsis. Front Immunol.

[9] Bush LM, Vazquez-Pertejo MT (2024). Nontyphoidal Salmonella infections. MSD Manual Professional Version.

[10] Sharma Sharma, Masood Masood, Kahlon Kahlon (2016). Streptococcus mitis bacteremia and endocarditis: an early sign in pre-cancerous colon polyps: 1366. Am J Gastroenterol.

[11] Cabezón G, de Miguel M, López J (2023). Contemporary clinical profile of left-sided native valve infective endocarditis: Influence of the causative microorganism. J Clin Med.

[12] Chambers HF, Bayer AS (2020). Native-valve infective endocarditis. N Engl J Med.

[13] Khan MZ, Munir MB, Khan MU, Khan SU, Benjamin MM, Balla S (2020). Contemporary trends in native valve infective endocarditis in United States (from the National Inpatient Sample Database). Am J Cardiol.

[14] Nappi F (2024). Native infective endocarditis: a state-of-the-art-review. Microorganisms.

